# Deep-learning map segmentation for protein X-ray crystallographic structure determination

**DOI:** 10.1107/S2059798324005217

**Published:** 2024-06-27

**Authors:** Pavol Skubák

**Affiliations:** a Leiden University Medical Center 2333 ZALeiden The Netherlands; University of Cambridge, United Kingdom

**Keywords:** computational modelling, molecular crystals, structure determination, experimental phasing, protein structure, macromolecular X-ray crystallography, density modification, single-wavelength anomalous diffraction, convolutional neural networks, deep learning

## Abstract

When solving a structure of a protein from single-wavelength anomalous diffraction X-ray data, the initial phases obtained by phasing from an anomalously scattering substructure usually need to be improved by an iterated electron-density modification. In this manuscript, the use of convolutional neural networks (CNNs) for segmentation of the initial experimental phasing electron-density maps is proposed. The results reported demonstrate that a CNN with U-net architecture, trained on several thousands of electron-density maps generated mainly using X-ray data from the Protein Data Bank in a supervised learning, can improve current density-modification methods.

## Abstract 2

Electron-density map segmentation into protein and solvent regions using deep neural networks improves density modification in X-ray structure solution from experimental phases.

## 1. Introduction

Density modification is a crucial step in the determination of macromolecular crystal structures by experimental phasing from the X-ray anomalous signal. The density-modification procedure aims to improve an initial electron-density map, obtained by phasing from a previously determined anomalous substructure, by incorporating prior information into the map. The prior information consists of features that are expected to be present in a well resolved electron-density map: flatness of the solvent regions (Wang, 1985 ▸), similarity of regions related by noncrystallographic symmetry, if present (Bricogne, 1974 ▸), or similarity of the density histogram to histograms of electron-density maps of deposited proteins (Lunin, 1988 ▸; Zhang & Main, 1990 ▸).

A partitioning of the electron-density map into protein and solvent regions, usually denoted as a ‘solvent mask’ or a ‘molecular envelope’, is typically used by the solvent-flattening and histogram-matching algorithms. In early applications, this partitioning was performed using local averaging of the density (Wang, 1985 ▸; Leslie, 1987 ▸). Later, solvent-mask determination using a local variance of the density (Abrahams & Leslie, 1996 ▸) was found to provide better results and has remained the state-of-the-art method for solvent-mask determination in classical density modification until today, as implemented, for example, in *Parrot* (Cowtan, 2010 ▸). The method of local variance has also recently been found to provide the best results in density modification for *ab initio*X-ray data phasing with large solvent contents (Kingston & Millane, 2022 ▸). The solvent content is an important parameter in the density-modification procedure and is traditionally estimated by estimation of the Matthews coefficient (Matthews, 1968 ▸), which was later updated using statistical inferences based on data from the PDB (Kantardjieff & Rupp, 2003 ▸; Weichenberger & Rupp, 2014 ▸).

In this manuscript, I propose density-map partitioning into solvent and protein regions using supervised deep learning with a 3D convolutional neural network (CNN) and its use for solvent-content parameter estimation. Although deep learning has revolutionized *ab initio* protein structure prediction using tools such as *AlphaFold*2 (Jumper *et al.*, 2021 ▸) and *RoseTTAFold* (Baek *et al.*, 2021 ▸), the number of deep-learning applications in the X-ray protein structure-determination methodology remains rather limited. Several CNN or attention-based architectures have been implemented for the prediction of protein crystallization propensity and for crystallization monitoring (see, for example, Yann & Tang, 2016 ▸; Khurana *et al.*, 2018 ▸; Elbasir *et al.*, 2020 ▸; Jin *et al.*, 2021 ▸; Wang *et al.*, 2023 ▸). A deep CNN has been implemented for the classification of 2D X-ray diffraction images (Ke *et al.*, 2018 ▸; Souza *et al.*, 2019 ▸). Miyaguchi *et al.* (2021 ▸) used a 3D CNN to predict local electron-density map correlations. Godo *et al.* (2022 ▸) have suggested the use of a CNN-based approach for model building, and Chojnowski *et al.* (2022 ▸) used deep learning for sequence prediction from the electron density. A multilayer CNN has been suggested for *ab initio*X-ray data phasing from Patterson maps, with promising results obtained on synthetic data for small peptide fragments (Pan *et al.*, 2023 ▸). A detailed overview of existing machine-learning applications in X-ray methodology, including ‘shallow’ machine learning, can be found in Matinyan *et al.* (2024 ▸). Furthermore, there have been numerous successful applications of 3D image segmentation using a deep CNN in other fields, such as medical imaging (see, for example, Chen *et al.*, 2020 ▸; Saood & Hatem, 2021 ▸).

## 2. Methods

### 2.1. Data preparation

The Protein Data Bank (PDB; Berman *et al.*, 2000 ▸) was searched using a PDBe REST API query by modifying the scripts provided by Bond & Cowtan (2022 ▸). The query asked for protein-containing data sets solved by X-ray crystallo­graphy with either the phasing method or the structure-solution method specified as single-wavelength anomalous diffraction (SAD), excluding data sets that were already present in the local collection. To the query date (20 December 2022), 10 891 items satisfying these criteria were returned by the API. After the exclusion of data sets for which the anomalous data had not been deposited, data sets for which the cell in the deposited structure differed considerably from the cell in the deposited data and data sets for which the anomalous signal was considered to be too weak, 2544 data sets remained (Table 1 ▸).

A cell was considered to be considerably different if a relative cell-axis difference was 3% or larger or if an angle difference was 3° or larger. The anomalous signal was considered to be too weak if the average phase difference between the phases constructed from the deposited protein model and the phases provided by *REFMAC*5 (Nicholls *et al.*, 2018 ▸) phasing from the deposited anomalously scattering substructure was larger than 85° (random phases correspond to an average phase difference of approximately 90°), or if the correlation of the estimated *E*-values with the deposited substructure *E*-values in the lowest resolution shell was 0.1 or worse. The correlation of the estimated *E*-values with the deposited substructure *E*-values was calculated in the same way as reported in Pannu & Skubák (2023 ▸): the deposited substructure *E*-values were calculated by *ECALC* (Ian Tickle, unpublished work) from the amplitudes provided by *REFMAC*5 for the deposited anomalously scattering sub­structure and their correlation with the estimated *E*-values was calculated using the *SFTOOLS* utility (Bart Hazes, unpublished work), which calculated the correlations in 20 resolution bins. Typically, structures with an anomalous signal that was too weak were originally solved by SAD phasing, but only the native data set without a significant anomalous signal was deposited in the PDB.

For each of the 2544 data sets, a complete *Crank*2 (Skubák & Pannu, 2013 ▸) SAD structure-solution run was performed using the default pipeline and default parameters: *SHELXC*/*D* (Schneider & Sheldrick, 2002 ▸) was used for anomalous substructure determination and *REFMAC*5 (Nicholls *et al.*, 2018 ▸), *Parrot* (Cowtan, 2010 ▸), *Buccaneer* (Cowtan, 2008 ▸) and *SHELXE* (Usón & Sheldrick, 2018 ▸) were used in the subsequent combined phasing, density modification and model building. The programs were used in versions corresponding to *CCP*4 (Agirre *et al.*, 2023 ▸) 8.0.008, except for *Crank*2, where a more recent version, 2.0.330, was used, and a bugfix in *REFMAC*5 implemented by me was used to prevent the program from crashing for very large data sets. The input to *Crank*2 consisted of the SAD data set (including information about the unit cell and space group) and protein sequence downloaded from the PDB and specification of the X-ray wavelength and the main anomalously scattering atom type, with anomalous scattering coefficients automatically derived from the wavelength by *Crank*2. Automatic heuristics were used to set the main anomalous scatterer from the atom types present in the PDB deposition, manually checked and potentially corrected for data sets where issues or bad performance were observed.

The default *Crank*2 pipeline built a model with an *R*_free_ smaller than or equal to 0.45 for 2163 data sets. For another 117 data sets the substructure was ‘correctly’ determined but the *R*_free_ was larger than 0.45; 107 of these also had a phase error after phasing of better than 85°. The anomalous sub­structure was considered to be ‘correctly determined’ if at least one third of atoms in the final anomalous substructure had a matching atom (within 2 Å distance) in the obtained substructure after transformation by *SITCOM* (Dall’Antonia & Schneider, 2006 ▸).

The remaining 264 data sets for which the *R*_free_ was larger than 0.45 and the substructure was not obtained by the default *Crank*2 pipeline were inputted into an ‘advanced’ *Crank*2 pipeline, which used *Afro* (Pannu & Skubák, 2023 ▸) for the calculation of multivariate normalized anomalous substructure amplitudes (denoted as *E*-values) and *PRASA* (Skubák, 2018 ▸) for substructure determination. Furthermore, in some cases multiple runs were performed manually adjusting substructure-determination parameters such as high-resolution cutoff ranges, number of trials, number of peaks or their special positions. Using this approach, the substructures of another 194 data sets were found, of which 141 led to a built model with *R*_free_ ≤ 0.45.

Finally, 70 data sets for which the substructure was not obtained were inputted into phasing from the deposited substructure. Furthermore, a further 23 data sets for which the substructure was obtained but led to a phase error worse than 85° (ten from the default pipeline and 13 from the advanced pipeline) after the initial phasing were also phased from the deposited substructure. Phasing from the deposited substructures was performed in the same way as phasing from determined substructures, *i.e* using the program *REFMAC*5, which included refinement of the substructure.

Another 266 data sets were added from a local database of data sets, composed of 159 data sets from Skubák (2018 ▸) for which the substructure was obtained and 107 JCSG data sets (Elsliger *et al.*, 2010 ▸) phased from the final substructure. Thus, a total of 2810 data sets were used for training and validation of the neural network. A summary of the used data sets is provided in Table 2 ▸.

Approximately 5% of the data sets were randomly assigned to a validation set. The structure of the validation set from the preparation point of view is shown in Table 3 ▸. In total, 137 data sets were in the validation set and the remaining 2673 data sets were in the training set.

In all of the preparation runs, initial phasing from the determined or the deposited anomalous substructure was performed using the SAD function implemented in *REFMAC*5 (Skubák *et al.*, 2004 ▸). Estimates of amplitudes and phases after the initial phasing calculated by the SAD function (FB and PHIB) were converted to phasing-map grids using a Shannon sampling rate corresponding to high resolution of the data, performed by the Python version of the GEMMI library for structural biology (Wojdyr, 2022 ▸). The phasing-map grids were used for the generation of input to the neural network model.

### 2.2. Model and training

The input to the binary classification neural network model is a vector of 48 × 48 × 48 windows from the phasing-map grids. The 48 × 48 × 48 input windows were randomly sampled from all of the prepared phasing-map grids. Since the sampling was random, their potential overlaps were not excluded and the unit cell did not have to be completely filled. Furthermore, data augmentation was used by flipping around a random number of unit-cell axes and rotating by 90° in a random direction to each of the input windows. In total, 36 462 training windows and 1895 validation windows were used, all of which were generated by random sampling and augmentation on the fly in each epoch. The GEMMI library was used for manipulation of the maps and the data sets.

The neural network model is a 3D convolutional U-net (Ronneberger *et al.*, 2015 ▸) using residual connections between layers (He *et al.*, 2016 ▸), implemented in the frameworks of *TensorFlow* (Abadi *et al.*, 2015 ▸) with *Keras* (Chollet, 2015 ▸). The U-net architecture is composed of four encoding convolution blocks followed by three decoding (upsampling) blocks and a final 3D convolution layer for the reduction of the number of filters to the number of classes. The output is a vector of 48 × 48 × 48 binary windows containing a classification of each window point as 0 or 1, corresponding to protein or solvent, respectively. The training and validation are performed against 48 × 48 × 48 windows from the ‘true’ solvent masks. The ‘true’ solvent masks were obtained by solvent/protein masking of the deposited protein models using the GEMMI framework, with its parametrization set to the van der Waals atomic radii set and the parameters for small solvent islands removal kept at their default values.

Each encoding convolution block consists of two 3D convolution layers with 3 × 3 × 3 kernels, both followed by batch normalization and ReLU activation. In the first three convolution blocks, a max pooling layer with a kernel size of 2 × 2 × 2 follows, reducing the dimensions by a factor of 2. The number of filters is set to 24 at the input to the first encoding block and is doubled in each of the following three encoding blocks.

Each decoding block starts with a transposed 3D convolution layer (also called a deconvolution layer) with a 3 × 3 × 3 kernel and a stride of 2. It is then followed by a concatenation of its output with the output of a corresponding (*i.e.* providing output with the same dimensions) encoding convolution block, thus creating the residual connections between the encoding and decoding blocks. Finally, the concatenation is followed by a 3D convolution layer with a 3 × 3 × 3 kernel, batch normalization and ReLu activation. Thus, the number of filters is halved at each decoding block, reaching the initial value of 24 after the last decoding block. The final 3D convolution layer uses a kernel of size 1 × 1 × 1 and reduces the number of filters to the number of classes, which is set to 2 for classification into a solvent class and a protein class.

Every 3D convolution layer in the model is used with ‘same’ padding to preserve dimensionality and the ‘he_normal’ kernel initializer. Sparse categorical cross-entropy was used as a loss function and the *Adam* algorithm (Kingma & Ba, 2014 ▸) with a learning rate of 0.0001 was used as a minimizer, both as implemented in the *Keras* framework. Training was performed locally on an NVIDIA Geforce RTX 3080 12G graphical card using the CUDA library (Vingelmann & Fitzek, 2020 ▸).

### 2.3. Implementation and testing

The segmentation of phasing maps into solvent and protein regions by a deep neural network model was implemented and tested for density modification within the *Crank*2 suite. The program *Parrot* was modified to accept and use the solvent masks predicted by the neural network model as its input. Although the U-net model was trained using density maps immediately after the initial phasing, it turned out that it was also useful for the segmentation of biased maps within the density-modification recycling. Thus, the implementation also uses the U-net map segmentation for subsequent density-modification cycles, not only for the first cycle. For each map-segmentation prediction, the input electron-density map is cut into 48 × 48 × 48 windows without overlaps (except for possible overlaps at the unit-cell edges, if the cell dimensions are not divisible by 48) and the 48 × 48 × 48 binary predictions outputted by the U-net model are then put together, providing the actual density-map segmentation.

Testing of the new *Crank*2 algorithm using U-net map segmentation for density modification was performed on the validation set of 137 SAD data sets (Table 3 ▸). It was performed using *CCP*4 8.0.016 and *Crank*2 2.0.342, with *Parrot* modified to use the solvent masks from the U-net map segmentation as mentioned before. For each data set, *Crank*2 was run from the substructure determined within the data preparation or from the deposited substructure to protein model building. The combined model-building algorithm (Skubák & Pannu, 2013 ▸) was used, with its density-modification part using the solvent masks from map segmentation. The testing compares the results of the *Crank*2 pipeline using the density modification by U-net map segmentation against the same pipeline with the ‘current’ density modification (as implemented in *Crank*2 2.0.342), with all other algorithms and parameters being the same.

The U-net map-segmentation output can be also used to estimate the overall crystal solvent content. The ‘current’ *Crank*2 density modification solely uses the Weichenberger & Rupp (2014 ▸) fit function Matthews estimation of solvent content. The density modification using U-net segmentation also takes into account a solvent-content estimate from the U-net segmentation in cases where this estimate is significantly different from the Matthews estimate (see equation 2 below).

Estimation of the solvent-content parameter is only performed in the initial cycle of density modification, *i.e.* using the map immediately after phasing, and the parameter is fixed throughout the following cycles, since it turned out that its re-estimation from the biased density-modification maps led to worse results. The expected value of the solvent content from map segmentation can be calculated as 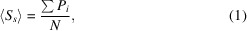
where *P*_*i*_ is the probability of the *i*th point in the unit-cell grid being solvent, as outputted by the neural network model, and *N* is the total number of points in the unit-cell grid. To save time, symmetry is taken into account and the summation is restricted to the asymmetric unit grid.

The expected solvent content from the map-segmentation prediction (equation 1) is then compared and combined with the Matthews coefficient prediction. The Matthews coefficient prediction provides a vector of solvent contents corresponding to different numbers of monomers in the asymmetric unit, together with their implied probabilities. If a high-probability Matthews solvent-content estimate consistent with the map-segmentation estimate exists, the Matthews prediction solvent-content value is used in density modification. If a less consistent match is found a combination of both estimates is used, and in the case of inconsistency only the expected value from the map segmentation (equation 1) is used. 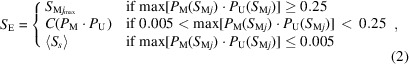
where *S*_E_ is the solvent-content estimate using information from both the U-net map segmentation and the Matthews estimation, *S*_M*j*_ denotes the solvent content corresponding to the *j*th Matthews coefficient with nonzero probability, *j*_max_ denotes the index of the Matthews coefficient with the largest probability, *P*_M_(*S*_M*j*_) is the probability of *S*_M*j*_ from the Matthews prediction, *P*_U_(*S*_M*j*_) is the probability of *S*_M*j*_ from the U-net model prediction and *C*(*P*_M_ · *P*_U_) denotes solvent corresponding to the centroid of the kernel of a probability distribution obtained by conflation (*i.e.* the product) of *P*_M_ and *P*_U_. *P*_M_ is defined on 〈0, 1〉 as a linear interpolation between all of the *P*_M_(*S*_M*j*_) points and the edge points (0, 1), for which it is assumed that *P*_M_(0) = 0 and *P*_M_(1) = 0. Since it is difficult to obtain *P*_U_ theoretically from the outputted *P*_*i*_ values (an assumption of independence of the *P*_*i*_ values is not justified as they are strongly intrinsically related), an empirical probability distribution *P*_U_ was constructed. At first, for each data set in the training set, a logit cutoff *l*_c_ was determined that led to the ‘true’ solvent content by setting all of the grid points with log(*P*_*i*_/(1 − *P*_*i*_)) > *l*_c_ (*i.e.* if the logit of *P*_*i*_ is larger than the cutoff logit) to solvent. Then, using all of the data sets in the training set, a histogram was created, counting in bins the number of data sets for which *l*_c_ belonged to a bin logit interval. Finally, from the histogram, the empirical bin-based probability distribution *P*_U_ was constructed by normalization to 1.

## 3. Results and discussion

Many hyperparameter and architecture adjustments were tested for U-net training and were evaluated against the accuracy metric as implemented in *Keras*. The architecture reported in Section 2 was eventually chosen; although some architecture or parametrization changes provided comparable accuracy results, the reported architecture with approximately three million trainable parameters appears to be close to a ‘sweet spot’ where adding more complexity barely improves the accuracy further, while reducing the complexity leads to worse results.

A total of 100 epochs were run with the reported architecture and parametrization using a batch size of 32. Due to a relatively large number of input data and data augmentation, overfitting was not a major problem. Yet, since the validation accuracy stopped improving after approximately 60 epochs at approximately 0.837 and the working accuracy kept slowly increasing (from 0.845 around epoch 60 to 0.847 at epoch 100), the model trained in epoch 57 was considered to be final and was used in subsequent density-modification testing on the validation set of data sets. The precision, recall, specificity and F1 score of the solvent classification on the validation set were all close to the accuracy value (0.839, 0.854, 0.818 and 0.846, respectively).

**Figure 1:**
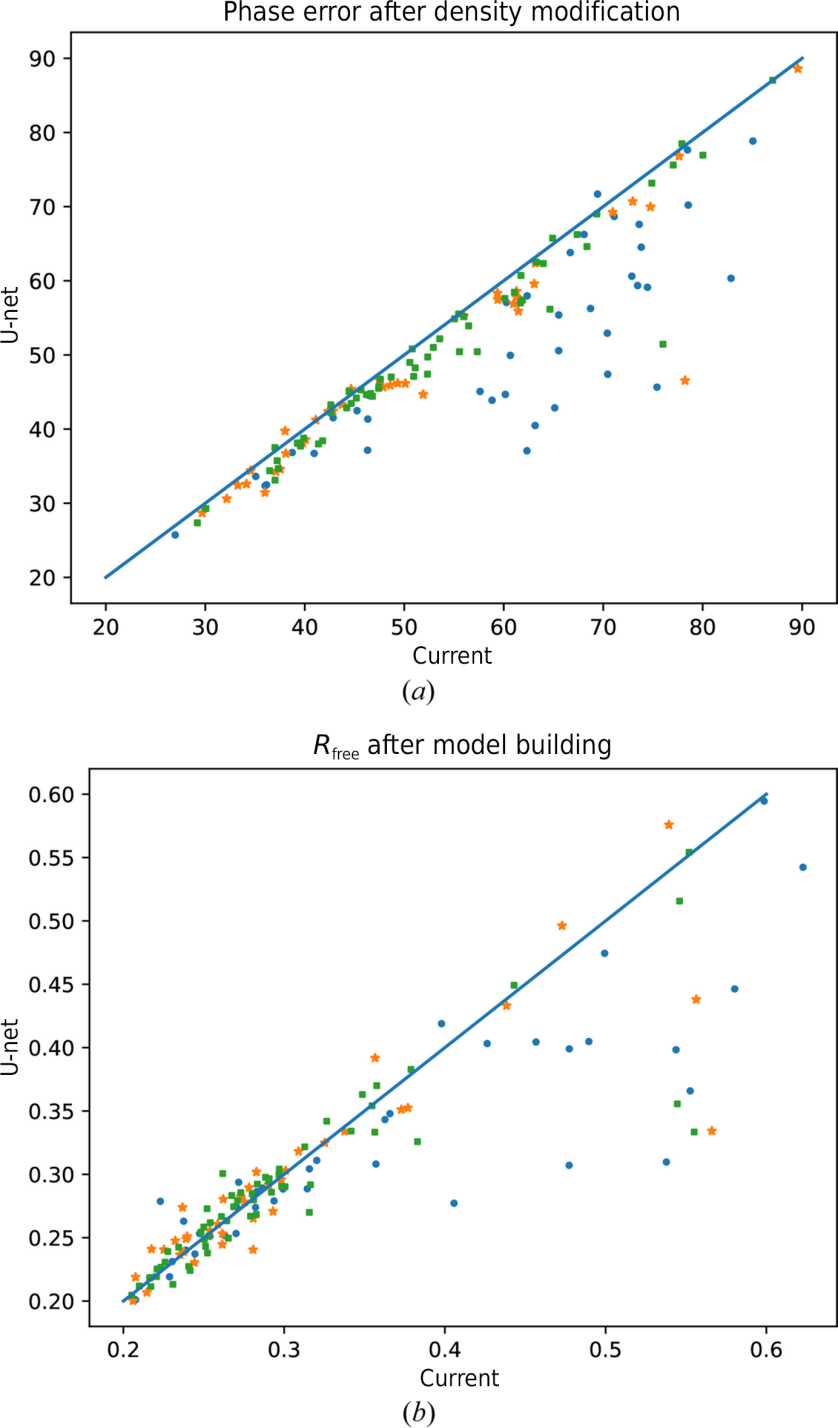
(*a*) Phase error after density modification. (*b*) *R*_free_ after model building using the current methods (*x* axis) and using deep-learning map segmentation (*y* axis) for each of the 137 data sets in the validation set. The data sets for which the inputted solvent content to density modification using both methods was equal (absolute value of the difference smaller than 0.01) are displayed as green squares, modest differences in the determined solvent content (absolute value of the difference between 0.01 and 0.1) are displayed as orange stars and large differences (absolute value of the difference of 0.1 or larger) are displayed as blue dots.

As Fig. 1 ▸(*a*) shows, the phase errors after density modification were substantially reduced overall when the U-net map segmentation was used to generate solvent masks for density modification. The phase error after density modification was worse than the current methods for approximately 9% of the tested data sets (12 out of 137) and better for the remaining 91% of the data sets. The regressions were typically tiny, with less than 1° of phase error. If we consider phase differences of less than 1° as approximately the same performance, then a phase-error improvement was observed for 105 data sets, a regression for two data sets and approximately the same result for the remaining 30 data sets.

The largest phase-error improvements were typically obtained for data sets where the default Matthews coefficient estimation provided a substantially different solvent-content estimate from the solvent-content estimate also utilizing the U-net prediction, proving that information from the U-net segmentation of experimental phasing maps can reliably be used for an improved solvent-content estimation. However, it should be noted that although the solvent-content estimation is substantially improved, it still does not guarantee an optimal solvent-content value for density modification. This can be demonstrated by the largest phase-error regression (2°), which was obtained for the data set with PDB code 5v63: the Matthews coefficient estimate of 0.4 turned out to provide slightly better density-modification results (using either solvent masks generated by the current methods or by the U-net) than the estimate of 0.22 obtained from the centroid of the conflated distributions, even though the solvent content obtained from the deposited model was 0.26, *i.e.* closer to the centroid estimate.

However, Fig. 1 ▸(*a*) also shows that the improvements were not limited to an improved solvent-content estimate: density modification also provided consistently better results when using the U-net-generated masks over the masks generated by the current methods in cases where the solvent-content estimate was the same. While an incorrect default solvent-content estimate could be curated by manual or automated testing of various solvent-content values, the improved prediction of solvent masks shows that the new method can significantly enhance the experimental phasing structure solution in general.

As Table 3 ▸ indicates, models for the majority of the validation data sets were already successfully built using the current methods. Since the new methods described in this paper provide density-modification enhancements, it is not surprising to observe that the good-quality models obtained using the current pipeline could not be further improved: as Fig. 1 ▸(*b*) demonstrates, the data sets for which an *R*_free_ better than approximately 0.35 was obtained using the current pipeline end up with a similar *R*_free_ when the U-net enhancements are used. However, large improvements were observed for partial models and even for data sets where no sensible model was built using the current methods with the default parametrization, including several data sets for which the solvent-content estimate was the same as when using the current methods. These results demonstrate that automated structure solution from experimental phases can be considerably improved by the use of neural network map segmentation.

The successful use of deep neural networks for electron-density map segmentation in experimental phasing density modification, as demonstrated by this paper, suggests further application opportunities in X-ray protein structure-solution methods. Density modification is also used in some protocols after molecular replacement of PDB or *AlphaFold*2 models; for example, to reduce the model bias when rebuilding the molecular-replacement model at lower resolutions. Furthermore, density modification is essential for *ab initio*X-ray protein structure solution using large solvent contents (Kingston & Millane, 2022 ▸). For both of these potential applications, it is unclear whether the reported U-net model for map segmentation would be sufficiently generic and robust, or whether retraining of the model against the specific data and possibly with a different architecture or hyper­parameters would be needed. Another density-modification improvement may be achieved by implementing a model that does not output the segmentation of the density map but rather the actual density modification itself. The experimental phasing-map segmentation and its confidence may also be useful for estimation of the quality of the phasing map and the related problems of the prediction of substructure-determination success and model-building success from the initial phasing map. Finally, an extended multiclass electron-density map segmentation (including classes such as missing or incorrect ligands, waters, ions *etc.*) may also be useful in the final stages of structure solution and its validation.

## Figures and Tables

**Table 1:** Numbers of SAD data sets downloaded from the PDB, excluded and used for training The exclusions were performed consecutively in the order of the rows (for example, if no anomalous data were present for a data set it was excluded and no other exclusion criteria were evaluated for that data set).

Total downloaded from the PDB	10891
Excluded due to no anomalous data deposited	7612
Excluded due to a different cell for the data and model	3
Excluded due to no or unusable anomalous signal	732
Total used from the PDB after exclusions	2544

**Table 2:** Preparation of data sets for training, validation and testing

No. of data sets	Downloaded from the PDB			Local database	
Substructure used	Determined		Deposited	Determined	Deposited
Pipeline used	Default	Advanced		Advanced	
Model built†	2163	141	n.a.	154	n.a.
Model not built	107	40	93	5	107

† *R*_free_ ≤ 0.45.

**Table 3:** Data sets used for validation

No. of data sets	Downloaded from the PDB			Local database	
Substructure used	Determined		Deposited	Determined	Deposited
Pipeline used	Default	Advanced		Advanced	
Model built†	98	11	n.a.	9	n.a.
Model not built	8	2	4	0	5

† *R*_free_ ≤ 0.45.
